# Tetraploidy Confers Superior *in vitro* Water-Stress Tolerance to the Fig Tree (*Ficus carica*) by Reinforcing Hormonal, Physiological, and Biochemical Defensive Systems

**DOI:** 10.3389/fpls.2021.796215

**Published:** 2022-01-28

**Authors:** Ruhollah Abdolinejad, Akhtar Shekafandeh

**Affiliations:** Department of Horticultural Science, College of Agriculture, Shiraz University, Shiraz, Iran

**Keywords:** fig tree, synthetic tetraploids, *in vitro* water stress, superior drought tolerance, hormonal and biochemical investigations

## Abstract

The fig tree is a well-adapted and promising fruit tree for sustainable production in arid and semi-arid areas worldwide. Recently, Iran’s dryland fig orchards have been severely damaged due to prolonged severe and consecutive drought periods. As emphasized in many studies, ploidy manipulated plants have a significantly enhanced drought tolerance. In the current study, we compared the induced autotetraploid explants of two fig cultivars (‘Sabz’ and ‘Torsh’) with their diploid control plants for their water stress tolerance under *in vitro* conditions using different polyethylene glycol (PEG) concentrations (0, 5, 10, 15, 20, and 25%). After 14 days of implementing water stress treatments, the results revealed that both tetraploid genotypes survived at 20% PEG treatments. Only ‘Sabz’ tetraploid explants survived at 25% PEG treatment, while both diploid control genotypes could tolerate water stress intensity only until 15% PEG treatment. The results also demonstrated that the tetraploid explants significantly had a higher growth rate, more leaf numbers, and greater fresh and dry weights than their diploid control plants. Under 15% PEG treatment, both tetraploid genotypes could maintain their relative water content (RWC) at a low-risk level (80–85%), while the RWC of both diploid genotypes drastically declined to 55–62%. The ion leakage percentage also was significantly lower in tetraploid explants at 15% PEG treatment. According to the results, these superiorities could be attributed to higher levels of stress response hormones including abscisic acid, salicylic acid, and jasmonic acid at different PEG treatments, the robust osmotic adjustment by significantly increased total soluble sugar (TSS), proline, and glycine betaine contents, and augmented enzymatic defense system including significantly increased superoxide dismutase (SOD), catalase (CAT), ascorbate peroxidase (APX), and glutathione peroxidase (GPX) activities in tetraploid genotypes, compared to their diploid control genotypes. Consequently, the current study results demonstrated that the ‘Sabz’ tetraploid genotype had a significantly higher water stress tolerance than other tested genotypes.

## Introduction

With the intensifying adverse effects of climate change, water stress has become a worldwide crisis and threat to agricultural development, especially in arid and semi-arid areas (Rai et al., 2011). About 41% of the Earth’s terrestrial surface is considered arid lands, and it is predicted to increase from 11 to 23% by the late 21st century (Leng et al., 2020).

The fig (*Ficus carica* L.) is a fruit tree from the Moraceae family, well-adapted to the arid and semi-arid areas worldwide, including Mediterranean regions and the Middle East, due to its great tolerance to harsh climates (Flaishman et al., 2008; Falistocco, 2009). For instance, fig orchards have been established throughout Iran, and over 84% of the orchards (51,000 ha from all 60,250 ha) are in dryland (without or very little precipitation during the growing season), where the average annual rainfall is between 250 and 400 mm (Roointan et al., 2016), with a dry and warm condition for five successive months during the growing season (Mesgaran et al., 2017). During the last two decades, severe and consecutive drought periods and shifting precipitation patterns influenced by climate change have seriously damaged fig production and industry in Iran. On the other hand, fig tree breeding is associated with complexities and barriers, including the existence of three different floral forms, a significant number of parthenocarpic cultivars, and the great heterozygosity rate (Mori et al., 2017). Therefore, developing efficient breeding methods to achieve highly drought-tolerant cultivars and/or rootstocks is necessary to maintain sustainable production and future demand (Flaishman et al., 2017; Abdolinejad et al., 2020).

Both cultivars investigated in this study are the figs sought after in Iran. ‘Sabz’ for its high-quality fresh and dry fruits and ‘Torsh’ for its unique fresh taste and flavor. ‘Sabz’ also is the most favorite fig cultivar for dryland fig production systems due to its good tolerance to drought conditions compared to other cultivars.

Polyploidy, or entire genome multiplication, is known as a major biological phenomenon with a crucial role in plant adaptation and diversification during the evolutionary periods (Ramsey and Schemske, 2002; Soltis et al., 2009; Fox et al., 2020). Because of the significant effects on whole-plant morphology and physiology, ploidy manipulation is an efficient breeding method that has been extensively used in crop improvement (Sattler et al., 2016; Ruiz et al., 2020). Due to the multiplied gene dosage, synthetic polyploids have enhanced the tolerance of many plant species to various abiotic stresses (Wei et al., 2019), including drought (Allario et al., 2013; De Souza et al., 2017; Oliveira et al., 2017; Wei et al., 2019; Rao et al., 2020), salt (Saleh et al., 2008; Wang et al., 2013; Ruiz et al., 2016b; Khalid et al., 2020), heat (Zhang et al., 2010), cold (Oustric et al., 2018), nutrient deficiency (Oustric et al., 2019), chromium toxicity (Balal et al., 2017), boron, and chloride excess (Ruiz et al., 2016a,b). Although the underlying molecular mechanisms by which neo-polyploids increase drought tolerance have not been clearly studied (Wei et al., 2019), the abscisic acid (ABA) signaling pathways, robust osmotic adjustment, and antioxidant defense system have been reported as the major mechanisms in this process. Allario et al. (2013) reported, compared to the diploid rootstocks, the over-expression of drought-responsive genes, particularly those involved in ABA biosynthesis and signaling pathway, had a key role in increasing drought tolerance of tetraploid rootstocks of *Citrus limonia*. Furthermore, Oliveira et al. (2017) discovered that the superiority of tetraploid seedlings of ‘Carrizo citrange’ for H_2_O_2_ scavenging is due to their higher *CAT2* expression, which could be the reason for their greater drought tolerance than diploid seedlings. Wei et al. (2019) also reported that tetraploid seedlings of *Poncirus trifoliata* exhibited remarkably higher drought tolerance compared with their diploid control plants. They explained that the strong reactive oxygen species (ROS) scavenging capacity due to over-expression of the genes related to the antioxidant defense system [peroxidase (POD) and superoxide dismutase (SOD)], robust osmotic adjustment (higher sugar accumulated), as well as lower levels of ROS accumulation were the major involved mechanisms. Rao et al. (2020) compared the transcriptomic analysis of diploid and tetraploid plants of *Lycium ruthenicum* subjected to severe drought stress. They reported that the superiority of tetraploid plants over diploids is attributed to their enhanced ABA biosynthesis (up to 78.4%) and more significant osmotic proteins’ expression.

In addition, morphological and anatomical alterations resulting from ploidy manipulation could also play an important role in plant drought tolerance. In this respect, polyploid plants have a larger stomata size but are less dense than diploid plants, leading to lower stomatal conductance, decreased transpiration rate, and improved photosynthetic efficiency under stressful conditions (Hias et al., 2017; Corneillie et al., 2019). Polyploidy could also effectively modify root hydraulic conductivity, which plays an important role in water status under drought stress. Polyploids may have thicker root cortex and suberin depositions, a well-known adaptive mechanism in plant systems to cope with water stress conditions, resulting in reduced transpiration rate and increased water-use efficiency (Doyle and Coate, 2019). These modifications were reported in tetraploid plants of *Citrus* spp. (Syvertsen et al., 2000; Ruiz et al., 2016a,b) and *Salix viminalis* (Dudits et al., 2016) and resulted in lower root hydraulic conductivity than their corresponding diploid plants (Ruiz et al., 2016b). In tetraploid plants of ‘Carrizo’ citrange, lower root hydraulic conductivity under drought stress give rise to maintaining gas exchange parameters and limiting water consumption, while diploid plants were greatly affected (Ruiz et al., 2016b; Oliveira et al., 2017).

Nowadays, implementing *in vitro* water stress experiments based on polyethylene glycol (PEG) treatments is frequently used to identify drought tolerance genotypes in higher plants (Kovalikova et al., 2020). PEG, an inert non-penetrating and non-ionic polymer with a high molecular weight (4000–8000 Da) and soluble in water, can decrease the water potential of the substrate by inducing osmotic stress without being phytotoxic (Emmerich and Hardegree, 1990; Ahmad et al., 2020) as it happens under field conditions.

The significant advantage of *in vitro* PEG-induced water stress is the possibility of precise control of any plant–environment interactions, which provide facilities to precisely study plant’s defensive strategies to water stress conditions and perform a rapid and convenient screening of drought tolerance genotypes. Thus, the results are reliable and reproducible (Peìrez-Clemente and Gomez-Cadenas, 2012). Compared to soil-based and hydroponic-based methods, it has certain fundamental advantages. Providing a constant and reproducible decline of substrate water potential that could not be obtained by the soil-based method and providing greater relevance to the natural field conditions due to performing on a solid substrate than hydroponic-based methods (Osmolovskaya et al., 2018). Despite the advantages, using high PEG concentrations may result in some limitations as it could interfere with solidifying the nutrient medium and increase the need for more agar. Also, high PEG concentration could enhance the viscosity of the medium and cause hypoxia (Osmolovskaya et al., 2018) which could be of concern in experimental design.

Climate change has intensified drought over the past decades in Iran and seriously damaged the dryland fig orchards. Therefore, using drought-tolerant rootstocks can be an effective approach to overcome this challenge. So, the main objective of this study was to compare the morphological, hormonal, physiological, and biochemical responses of tetraploid and diploid plants of two fig cultivars under induced drought tolerance and identify the most tolerant genotypes.

## Materials and Methods

### Plant Materials and Water Stress Treatments

**FIGURE 1:**
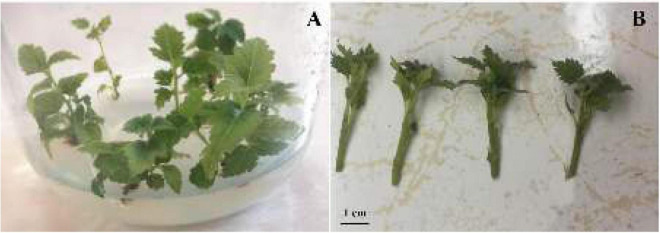
*In vitro* pre-culture of diploid and tetraploid explants on PGR-free MS medium as the explant source for water stress experiment **(A)**, and the isolated shoot tip explants for implementation of water stress treatments **(B)**.

*In vitro* tetraploid explants of two fig cultivars (‘Sabz’ and ‘Torsh’) were obtained from our previous work (Abdolinejad et al., 2021), and their diploid control explants (as four genotypes) were used in this study to compare their responses to different water stress treatments. The uniform 4 cm explants were obtained from shoot tips pre-cultured on PGR-free MS (Murashige and Skoog, 1962) medium with 3% sucrose and 0.8% agar (Figure 1). To implement water stress treatments, the selected explants were cultured on the same medium supplemented with various concentrations of 0 (control), 5 (mild), 10 (moderate), 15 (high), 20 (severe), and 25 (extreme) % (w/v) of PEG 6000. The media were sterilized by 20 min autoclaving at 121°C after their pH was adjusted to 5.8. Except for explants of control and mild treatments, all other explants were initially cultured on 5% PEG concentration and gradually transferred to the target treatments in 12 h intervals to avoid high-level water stress shock. PEG was added to the media after autoclaving through the 45-μ syringe filters when the media temperature dropped to 70–80°C. All cultures were kept in a growth chamber with 24 ± 1°C temperature, 16/8 h light/darkness, and a light intensity of 40 μmol m^–2^ s^–1^ for 14 days. At the end of the experiment (14th day), all the morphological, hormonal, physiological, and biochemical measurements were performed.

### Phytohormonal Analysis

The concentrations of three major stress response phytohormones, including ABA, salicylic acid (SA), and jasmonic acid (JA) in tetraploid and diploid explants, were determined according to Pan et al. (2010) with some modifications. Approximately 250 mg of leaf samples were taken from upper leaves, instantly transferred to liquid nitrogen, and then lyophilized. The samples were then ground to a fine powder in liquid nitrogen and extracted for their phytohormonal content by adding 0.5 ml of 1-propanol: distilled water: HCl (2:1:0.002 v:v:v) solution and shaking for 45 min at 3°C. Then, 1 ml of dichloromethane was added to the mixtures, and the samples were shaken for another 45 min at 3°C. The extraction solutions were centrifuged at 15,000 × *g* for 7 min. at 3°C. The lower phases (1 ml) were taken, immediately filtered (using 22 μm syringe filters), and transferred into the new tubes, then lyophilized and re-solubilized in 500 μl methanol [high performance liquid chromatography (HPLC) grade].

#### Ultra Performance Liquid Chromatography–Mass Spectrometer Analysis

The liquid chromatography–mass spectrometry (LC-MS) analysis was performed by an ACQUITY UPLC H-Class System device equipped with an ACQUITY QDa Mass Detector (Waters Corporation, Milford, MA, United States), with an analytical column C18 (2.1 mm × 50 mm, 1.7 μm; Waters Corporation). The flow rate was 0.5 ml/min, and injection volume of 5 μl, eluted with an isocratic mixture of 30% (v/v) HPLC-grade water and 70% (v/v) acetonitrile + 0.1% formic acid. Different concentrations of standard solutions (0.02–2000 ng/ml) for all phytohormones were prepared and used for plotting the standard curves. The data was presented as nanograms per gram lyophilized tissue (ng/g lyophilized tissue).

### The Survival Rate, Growth, and Proliferation Rate, and Morphological Measurements

The survival rate percentage of explants was calculated using the following formula:


$$Survivalrate\left(\%\right)=\left(\frac{N\,u\,m\,b\,e\,r\,o\,f\,s\,u\,r\,v\,i\,v\,e\,d\,e\,x\,p\,l\,a\,n\,t\,s}{T\,o\,t\,a\,l\,n\,u\,m\,b\,e\,r\,o\,f\,i\,n\,c\,u\,b\,a\,t\,e\,d\,e\,x\,p\,l\,a\,n\,t\,s}\right)\times 100$$

Shoot length was measured by a ruler and the average daily growth rate of explants was calculated as AGR (day^–1^) using the following formula:


$$\mathrm{Average}\,\mathrm{growth}\,\mathrm{rate}=\left(\frac{H\,2-H\,1}{T}\right)$$

where H1 = First measurement, H2 = Second measurement, and T = Time interval between two measurements.

For proliferation rate, only shoots larger than 5 mm were considered, and the rate was estimated using the following formula:


Proliferation⁢rate=⁢(Total⁢number⁢of⁢shoots⁢larger⁢than⁢ 5⁢mmTotal⁢number⁢of⁢incubated⁢explants)

Stem diameter was measured using a digital caliper. Leaf area was measured using a leaf area meter device (CRLA1, Iran). The fresh weight (FW) and dry weight (DW) of explants were measured using an analytical balance. For DW, the explants were oven-dried at 60°C for 48 h then weighed.

### Physiological Measurements

#### Relative Water Content

For measuring relative water content (RWC), leaf disk samples about 2 cm in diameter were provided, and their weight was initially measured. Then the samples were immersed in distilled water for 24 h to determine the turgid weight. Leaf DW were determined after oven-drying at 65°C for 48 h. RWC was estimated using the following formula:


$$RWC\left(\%\right)=\left(\frac{F\,r\,e\,s\,h\,w\,e\,i\,g\,h\,t-d\,r\,y\,w\,e\,i\,g\,h\,t}{T\,u\,r\,g\,i\,d\,w\,e\,i\,g\,h\,t-d\,r\,y\,w\,e\,i\,g\,h\,t}\right)\times 100$$

#### Ion Leakage

For estimating leaf ion leakage, the individual leaf samples were incubated in vials containing 10 ml of distilled water for 24 h at room temperature (22°C) on a rotary shaker (100 rpm). The initial electrical conductivity (EC1) was recorded. The samples were then autoclaved at 121°C for 20 min. After cooling the solutions, the second electrical conductivity (EC2) was recorded, and the electrolyte leakage was calculated following the formula:


$$EL\left(\%\right)=\left(\frac{E\,C\,1}{E\,C\,2}\right)\times 100$$

#### Photosynthetic Pigments Content

According to Roy et al. (2021), for estimation of the total chlorophyll and total carotenoid contents, approximately 100 mg of leaf samples were ground into fine powder in liquid nitrogen and homogenized with 10 ml of ethanol: acetone: distilled water (4.5:4.5:1) and kept at 4°C for 24 h. The mixtures were then centrifuged at 10,000 × *g* for 10 min at 4°C. Light absorbance of samples was recorded by a microplate spectrophotometer (Epoch, BioTek, United States) at 645 and 663 nm for total chlorophyll and 470 nm for total carotenoid contents. Data were presented as milligram per gram FW (mg g^–1^ FW).

#### Lignin Content

Lignin content was determined according to the acetyl bromide digestion method described by Iiyama and Wallis (1990). The leaf samples (100 mg) were ground into a fine powder, mixed, and heated with 20 ml distilled water at 65°C. The mixtures were passed through the 45-μ filters, and the residues were thoroughly washed with ethanol, chloroform–methanol (2:1), and acetone, then dried at 100°C for 5 min. Thereafter, 6 mg of dried samples were mixed to 5 ml of 25% acetyl bromide in a glacial acetic acid solution containing 100 μl of 70% perchloric acid and heated at 70°C for 30 min and then samples were quickly cooled on ice. The samples were treated with 5 ml of NaOH (2N) and glacial acetic acid. Light absorbance of samples was recorded by a spectrophotometer (Epoch, BioTek, United States) at 280 nm, and lignin content was presented as a percentage of the cell wall.

### Biochemical Measurements

#### Oxidative Markers

Further in experiment, 5 g of leaf samples from all genotypes and all treatments were collected. The samples were immediately transferred to liquid nitrogen, then ground into a fine powder, and stored at −80°C to use for the following measurements.

##### Hydrogen Peroxide (H_2_O_2_) Content

According to Sarker and Oba (2018), 200 mg of leaf fine powder was mixed with 1 ml of trichloroacetic acid (TCA) (0.1%) for a while and then centrifuged at 13,000 × *g* for 10 min at 4°C. Reaction mixture including 500 μl of supernatant, 500 μl of 100 mM potassium phosphate buffer (pH = 7), and 1 ml of 1 M potassium iodide was kept in darkness for 60 min. Light absorbance of samples was recorded by a spectrophotometer (Epoch, BioTek, United States) at 390 nm, and H_2_O_2_ concentration was presented as micromole H_2_O_2_ per gram FW (μmol g^–1^ FW).

##### Malondialdehyde Content

Lipid peroxidation was determined by measuring the malondialdehyde (MDA) concentration in leaf tissue according to Hodges et al. (1999). In brief, 100 mg of leaf fine powder was homogenized in 2 ml ethanol: distilled water (80% v/v) and centrifuged at 10,000 × *g* for 15 min. Then 1 ml of supernatant was mixed with 1 ml thiobarbituric acid (TBA) solution comprised of 20.0% (w/v) TCA and 0.01% butylated hydroxytoluene. The mixture was then heated at 95°C for 30 min and immediately cooled on ice and centrifuged at 7000 × *g* for 10 min. Light absorbance of samples was recorded by a spectrophotometer (Epoch, BioTek, United States) at 414, 532, and 600 nm. The MDA content was calculated using the following formula:


MDA= 6.45⁢(Abs⁢ 532-Abs⁢ 600)- 0.56⁢Abs⁢ 414

Data were presented as micromole MDA per gram FW (μmol g^–1^ FW).

#### Antioxidant Enzymes Measurements

To measure the activity of antioxidant enzymes, approximately 2 g of frozen powder (−80°C) was mixed with 3 ml of extraction buffer composed of potassium phosphate buffer (50 mM, pH 7.8), 100 μM EDTA, 0.2% triton x-100, and 2% polyvinylpolypyrrolidone. The mixture was centrifuged at 13,000 × *g* for 15 min, and the supernatant was transferred to a new tube and used for further analyses (Pan et al., 2006). All operations for antioxidant enzyme activity were performed at 3°C.

##### Superoxide Dismutase Activity

Superoxide dismutase activity was determined according to Manquián-Cerda et al. (2016) based on its ability to inhibit the photochemical reduction of nitroblue tetrazolium (NBT). The reaction mixture consists of 300 μl of enzyme extract, 400 μl potassium phosphate buffer (100 mM, pH 7.0), 10 μl EDTA (10 mM), 50 μl methionine (260 mM), 80 μl NBT (4.2 mM), and 170 μl riboflavin (130 μM). It was illuminated by a light intensity of 60 μmol m^–2^ s^–1^, provided by cool-white, fluorescent tubes for 15 min. Light absorbance of samples was recorded by a spectrophotometer (Epoch, BioTek, United States) at 560 nm, and SOD activity was presented as unit SOD activity per mg protein per minute (U mg^–1^ protein min^–1^).

##### Catalase Activity

For catalase (CAT) activity assessment, according to Chance and Maehly (1955), 15 μl of enzyme extract was added to the 2885 μl potassium phosphate buffer (50 mM, pH 7) and 100 μl hydrogen peroxide (10 mM), to a volume of 3 ml reaction mixture. Light absorbance of samples was recorded by a spectrophotometer (Epoch, BioTek, United States) at 240 nm, and CAT activity was determined by recording the decrease in optical absorbance resulting from the H_2_O_2_ decomposition rate for 1 min. CAT activity was presented as unit CAT activity per milligram protein per minute (U mg^–1^ protein min^–1^).

##### Ascorbate Peroxidase Activity

Ascorbate peroxidase (APX) activity was determined according to Nakano and Asada (1981). The reaction started by adding 20 μl of enzymatic extract to 1950 μl potassium phosphate buffer (50 mM, pH 0) containing 100 μM EDTA and 100 μM sodium ascorbate, and 30 μl hydrogen peroxide (2.5 mM). The decrease in light absorbance was recorded by a spectrophotometer (Epoch, BioTek, United States) at 290 nm, and the APX activity was presented as unit APX activity per milligram protein per minute (U mg^–1^ protein min^–1^).

##### Glutathione Peroxidase Activity

Glutathione peroxidase (GPX) activity was determined according to Ozden et al. (2009). GPX activity was assessed by measurements of the oxidation of guaiacol in the presence of hydrogen peroxide for 2 min in 20 s intervals. The reaction mixture contained 50 μl of guaiacol (20 mM), 2900 μl of potassium phosphate buffer (10 mM, pH 7), and 50 μl of enzyme extract, and the reaction started by adding 20 μl of H_2_O_2_ (40 mM). Light absorbance of samples was recorded by a spectrophotometer (Epoch, BioTek, United States) at 470 nm, and GPX activity was presented as unit GPX activity per milligram protein per minute (U mg^–1^ protein min^–1^).

#### Compatible Solutes Measurements

##### Total Soluble Sugars

The method described by Patade et al. (2012) was used for the estimation of leaf total soluble sugars (TSSs). In brief, 100 mg of lyophilized leaf tissue was first fine powder and extracted in 10 ml of ethanol: distilled water solution (8:2 v:v) and kept at room temperature for 30 min. The mixture was centrifuged at 10,000 × *g* for 15 min at 4°C. Then, the supernatant (1 ml) was mixed with 3 ml of Anthrone reagent (150 mg anthrone + 100 ml H_2_SO_4_ 72%) and placed in a water bath (100°C) for 15 min, then immediately cooled on ice. Finally, light absorbance was recorded by a microplate spectrophotometer (Epoch, BioTek, United States) at 620 nm. Glucose was used as a standard sample, and data were presented as milligram per gram DW (mg g^–1^ DW).

##### Proline Content

Free proline content was measured according to Bates et al. (1973). Leaf frozen powder (−80°C) was homogenized in 3% (w/v) sulfosalicylic acid and centrifuged at 7000 × *g* for 5 min. Then, 2 ml of supernatant was reacted with 2 ml acid ninhydrin and 2 ml of glacial acetic acid and kept at 100°C for 60 min in a water bath, then immediately cooled on ice. Four milliliters of toluene was added to the mixture and vortexed for 10 s, and kept at room temperature for a while. Two phases were formed, and the supernatant was used to determine free proline content. Light absorbance was recorded by a spectrophotometer (Epoch, BioTek, United States) at 520 nm, and proline content was presented as μmol proline g^–1^ leaf FW.

##### Glycine Betaine Content

The method of Patade et al. (2012) was used to determine the leaf glycine-betaine content. Leaf frozen powder (200 mg) was homogenized in 3 ml of distilled water and kept on a rotary shaker for 16 h at room temperature. The sample was then filtered (45 μm filters), and the extract was mixed equally with sulfuric acid (1:1, v:v) and placed on ice for 60 min. Moreover, 500 μl of extract reacted with 200 μl of iodine-potassium iodide (KI-I_2_) reagent and incubated for another 16 h at 4°C. The mixture was centrifuged at 10,000 × *g* for 5 min at 0°C, and the mixture was lyophilized. The per-iodide crystal formed was dissolved in 1 ml of 1,2-dichloroethane, and light absorbance was recorded by a spectrophotometer (Epoch, BioTek, United States) at 365 nm. The data was presented as μmol glycine-betaine g^–1^ leaf FW.

### Experiment Design and Statistical Analysis

An *in vitro* experiment was carried out based on a factorial experiment including 2 genotypes × 2 ploidy levels × 6 PEG concentrations in a completely randomized design (CRD) with 10 replications (glass jars) and 5 explants per replication. Hence, a total of 1200 explants were used. At the end of the experiment, four replications were used to measure the morphological, phytohormonal, physiological, and phytochemical parameters. Data were subjected to three-way ANOVA analysis using the SAS software version 9.4 (SAS Institute, United States). Although interactions of three-way were statistically significant, the results of two-way ANOVA analysis of ploidy level × PEG treatment within each cultivar were presented to emphasize the main research hypothesis. Means were compared using the LSD test at *P* < 0.05. Log transformation was performed, where zero was observed in the data.

## Results

Analysis of variance showed that the interaction effects between cultivar, Ploidy level, and PEG treatments were significant for all measured traits. Therefore, the presented results for all measured parameters indicate the interaction effects. Besides, 15% PEG treatment is identified as the critical water stress level in the present study due to the diploid genotypes not surviving in higher concentrations.

### Morphological Responses

**FIGURE 2:**
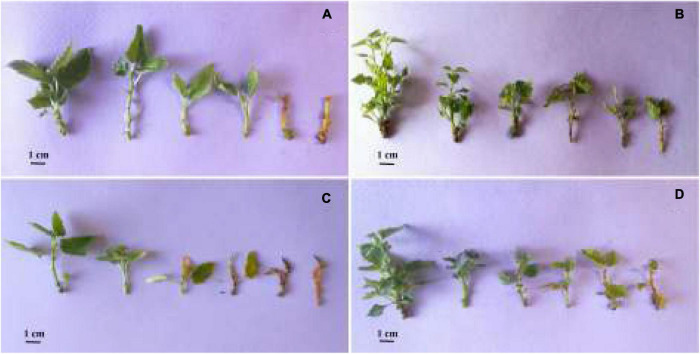
*In vitro* responses of diploid and tetraploid explants of ‘Sabz’ and ‘Torsh’ fig cultivars, 14 days after subjecting to different water stress treatments. In each section **(A–D)**, the explants belonged to PEG treatments of 0, 5, 10, 15, 20, and 25%, from left to right, respectively. **(A)** Diploid explants of ‘Sabz’ cultivar, **(B)** tetraploid explants of ‘Sabz’ cultivar, **(C)** diploid explants of ‘Torsh’ cultivar, and **(D)** tetraploid explants of ‘Torsh’ cultivar.

The results showed that the survival rate percentage of explants at 0, 5, and 10% PEG treatments was 100% for all genotypes. While the tetraploid genotypes had 100% survival at high water stress treatment (15% PEG), the survival rate of diploid genotypes declined to 80% for cultivar Sabz and 55% for cultivar Torsh. Furthermore, the tetraploid explants of ‘Sabz’ displayed a significantly higher survival rate (100%) than the tetraploid explants of ‘Torsh’ (45%) under severe water stress treatment (20% PEG) while as there were no surviving diploid explants at this level of water stress. Interestingly, the only surviving genotype under extreme water stress treatment (25% PEG) was tetraploid explants of ‘Sabz’, displaying a 70% survival rate (Table 1 and Figure 2).

**TABLE 1:** The effect of different PEG concentrations on survival rate, proliferation rate, and various morphological characteristics of tetraploid and diploid explants of ‘Sabz’ and ‘Torsh’ fig cultivars 14 days after subjecting to water stress treatments.

Cultivar	Ploidy level	PEG concentrations	Survival rate (%)	Proliferation rate	Shoot length (cm)	Growth rate (cm/d)	Stem diameter (mm)	Leaf number	Total leaf area (cm^2^)	Fresh weight (g)	Dry weight (g)
‘Sabz’	2×	0	100 a	3.1 b	7.02 b	0.272 b	4.52 a	6 cd	50.66 c	9.49 b	3.07 b
		5	100 a	1.12 d	5.1f d	0.145 c	3.24 d	4.5 ef	29.76 d	6.88 d	2.2 d
		10	100 a	0 e	3.7 e	0.045 de	2.48 f	3.2 fg	17.49 ef	5.21 f	1.92 e
		15	80 b	0 e	3.07 f	0.020 ef	2.03 g	1.5 h	7.61 gh	4.57 g	1.46 f
		20	0 d	–	–	–	–	–	–	–	–
		25	0 d	–	–	–	–	–	–	–	–
	4×	0	100 a	4.8 a	8.12 a	0.362 a	4.23 b	16 a	89.2 a	10.75 a	3.46 a
		5	100 a	2.8 c	6.35 c	0.232 b	3.72 c	12.75 b	67.95 b	8.15 c	2.63 c
		10	100 a	0 e	5.3 d	0.160 c	2.83 e	7.25 c	35.52 d	6.35 e	2.07 de
		15	100 a	0 e	5.27 d	0.155 c	2.43 f	4.75 de	19.8 e	6.31 e	2.03 de
		20	100 a	0 e	3.92 e	0.062 d	2.06 g	3 g	12.01 fg	3.38 h	1.24 fg
		25	70 c	0 e	3.05 f	0.002 f	1.83 h	1.5 h	4.82 hi	3.19 h	1.02 g
‘Torsh’	2×	0	100 a	3.3 b	7.05 b	0.285 b	4.22 a	5.75 c	56.16 a	10.37 a	3.34 a
		5	100 a	0 d	4.85 d	0.130 d	3.41 b	3.5 d	27.31 b	7.15 c	2.3 c
		10	100 a	0 d	3.12 f	0.007 f	2.83 d	2.25 e	11.48 cd	4.61 e	1.48 e
		15	55 b	0 d	3 f	0 f	2.31 f	1.25 e	4.24 de	3.21 f	1.03 f
		20	0 c	–	–	–	–	–	–	–	–
		25	0 c	–	–	–	–	–	–	–	–
	4×	0	100 a	4.6 a	7.52 a	0.320 a	4.28 a	10 a	56.15 a	9.79 b	3.15 b
		5	100 a	2.1 c	5.77 c	0.192 c	3.31 bc	8 b	34.49 b	7.27 c	2.34 c
		10	100 a	0 d	5.1 d	0.145 d	3.15 c	5 c	18.31 c	6.05 d	1.94 d
		15	100 a	0 d	4.85 d	0.127 d	2.62 e	2 e	6.86 de	4.50 e	1.44 e
		20	45 b	0 d	3.4 e	0.022 e	2.07 g	1.25 e	3.69 e	3.08 f	0.99 f
		25	0 c	–	–	–	–	–	–	–	–

*Means are present the ploidy level and PEG treatment effects tested by two-way ANOVA. Different letters in each column indicate significant differences at P < 0.05 using the LSD test.*

Water stress treatments significantly suppressed the proliferation of all genotypes, so that, shoot proliferation was only observed at the control and 5% PEG treatments for all genotypes. Accordingly, the highest proliferation rate (4.8 shoots per explant) was found in tetraploid explants of ‘Sabz’ in the control treatment, and the lowest proliferation rate (without any lateral branch) was found in all diploid and tetraploid explants at 10, 15, 20, and 25% PEG treatments.

Water stress treatments had a significant restrictive effect on all measured morphological features for all genotypes. According to the results, tetraploid explants of both cultivars were less affected by the detrimental effects of water stress treatments at 5, 10, and 15% PEG treatments than their diploid control plants. However, the most difference between tetraploid and diploid explants of both cultivars was observed at 15% PEG treatment, where all morphological indices in tetraploid genotypes were significantly higher. In addition, morphological indices were remarkably higher in tetraploid explants of ‘Sabz’ than tetraploid explants of ‘Torsh’ at the 20% PEG treatment. At 25% PEG treatment, only tetraploid explants of ‘Sabz’ survived (Table 1).

According to the results, the shoot length in tetraploid explants of ‘Sabz’ and ‘Torsh’ was significantly higher (1.7-fold and 1.6-fold, respectively) than their diploid control plants under 15% PEG treatments. Under 20% PEG treatment, tetraploid explants of ‘Sabz’ had significantly higher shoot length (13% higher) than the tetraploid explants of ‘Torsh’. Also, the shoot length of ‘Sabz’ tetraploids displayed a significant decrease at 25% PEG treatment (up to 22%) compared with 20% PEG treatment. A similar trend was observed for the growth rate of the tetraploid genotypes which were significantly higher than their diploid control plants at 15% PEG treatment (Table 1).

The results also demonstrated that with increasing PEG concentration, the stem diameter was notably decreased for all genotypes. However, compared to ‘Sabz’ and ‘Torsh’ diploid explants, their corresponding tetraploid explants displayed a significantly greater stem diameter under 15% PEG treatment (1.2-fold and 1.13-fold, respectively). There was no significant difference between tetraploid explants of ‘Sabz’ and ‘Torsh’ under 20% PEG treatment (Table 1).

As shown in Figure 2, tetraploid explants of both cultivars intrinsically have greater leaf numbers than their diploid control plants. The results revealed that with increasing water stress intensity, leaf number significantly declined in all genotypes. Both tetraploid genotypes had significantly greater leaf numbers at 5, 10, and 15% PEG treatments than their diploid control explants. In addition, the leaf number in ‘Sabz’ and ‘Torsh’ tetraploid explants was (3.1-fold and 1.6-fold, respectively) higher than their diploid control plants at 15% PEG treatment. At 20% PEG treatment ‘Sabz’ tetraploid explants had significantly greater leaf numbers (2.4-fold) than ‘Torsh’ tetraploid explants, and at 25% PEG treatment, ‘Sabz’ tetraploid explants showed a 50% decline in leaf number compared to 20% PEG treatment (Table 1).

Increased PEG concentrations considerably decreased the total leaf area for all genotypes compared to their control treatment. However, tetraploid genotypes showed a remarkably larger total leaf area (2.6-fold and 1.6-fold, in ‘Sabz’ and ‘Torsh’, respectively) at 15% PEG treatments than their diploid control plants. Under 20% PEG treatment, the total leaf area in tetraploid explants of ‘Sabz’ was significantly (up to threefold) greater than tetraploid explants of ‘Torsh’. The total leaf area in ‘Sabz’ tetraploids was 4.82 cm^2^ at 25% PEG treatment which showed a drastically decreased area (about 60%) than 20% PEG treatment (Table 1).

Fresh and dry weights of all genotypes significantly decreased by increasing PEG concentrations. Nevertheless, among all genotypes, the FW and DW of ‘Sabz’ tetraploid explants were significantly less affected by water stress treatments than the other genotypes. At 15% PEG treatment, ‘Sabz’ tetraploids displayed the maximum FW and DW (6.31 and 2.03 g, respectively) compared to the other genotypes. At 20% PEG treatment, there was no significant difference between ‘Sabz’ and ‘Torsh’ tetraploid explants in relation to the FW, however, ‘Sabz’ tetraploids displayed a significantly higher DW (1.24 g) than ‘Torsh’ tetraploids (0.99 g) (Table 1).

### Hormonal Responses

**FIGURE 3:**
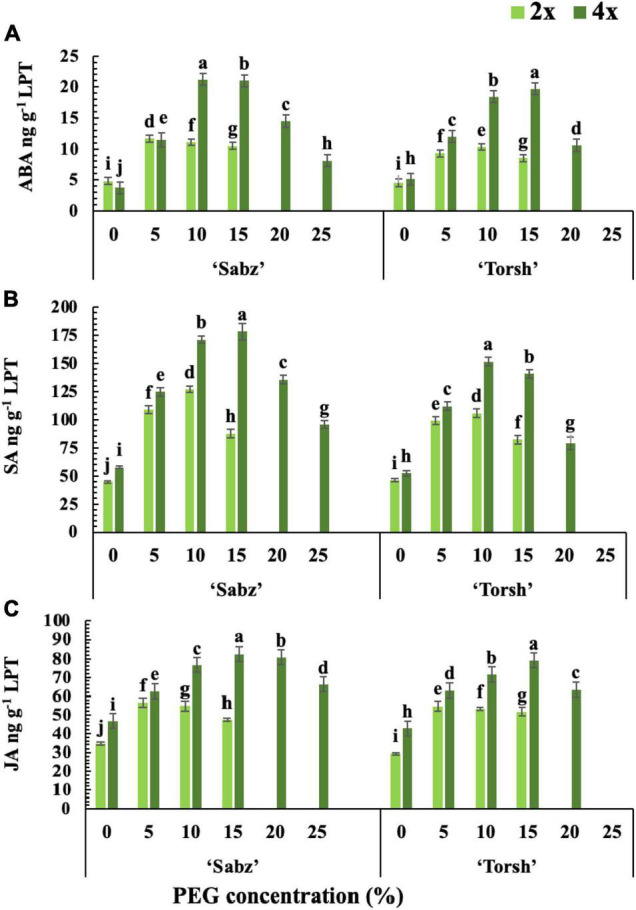
The cumulative biosynthesis amount of three main stress response phytohormones in tetraploid and diploid explants of ‘Sabz’ and ‘Torsh’ fig cultivars 14 days after subjecting to different water stress treatments. **(A)** Abscisic acid, **(B)** salicylic acid, and **(C)** jasmonic acid. LPT, lyophilized tissue. Means represent the ploidy level and PEG treatment effects tested by two-way ANOVA. Each value represents the means ± SE. Different letters indicate significant differences at *P* < 0.05 using the LSD test.

The results revealed that water stress treatments significantly increased the ABA, SA, and JA biosynthesis in all genotypes. However, hormonal biosynthesis and accumulation were significantly higher in both tetraploid genotypes than their diploid control explants (Figures 3A–C). ABA biosynthesis was 2-fold and 2.3-fold higher in ‘Sabz’ and ‘Torsh’ tetraploid genotypes, respectively, than their diploid control explants at 15% PEG treatment. At 20% PEG treatment, the ABA biosynthesis in ‘Sabz’ tetraploid explants was significantly higher (1.4-fold) than ‘Torsh’ tetraploid explants. As the only surviving genotype, the ABA content of ‘Sabz’ tetraploid explants was 8.12 ng/g LPT at 25% PEG treatment which showed a significant decrease than 20% PEG treatment (Figure 3A).

The SA and JA biosynthesis of tetraploid and diploid genotypes also displayed a similar increase trend with ABA at 10 and 15% PEG treatments. Tetraploid explants of ‘Sabz’ and ‘Torsh’ genotypes displayed significantly increased SA biosynthesis up to 2- and 1.7-fold at 15% PEG treatment, respectively, when compared to their diploid control plants. Under 20% PEG treatment, the SA biosynthesis was 1.7-fold higher in tetraploid explants of cultivar Sabz than tetraploid explants of cultivar Torsh. The SA content of ‘Sabz’ tetraploids at 25% PEG treatment (95.77 ng/g LPT) was decreased by 29.5% compared with its content at 20% PEG treatment (Figure 3B). JA biosynthesis also increased by 1.74-fold and 1.53 at 15% PEG treatment in tetraploid explants of cultivars Sabz and Torsh, respectively, compared to their diploid control explants. Furthermore, JA biosynthesis was 1.27-fold higher in tetraploid explants of cultivar Sabz than tetraploid explants of cultivar Torsh, and JA content in ‘Sabz’ tetraploid explants was 66.2 ng/g LPT at 25% PEG treatment, which showed an 18% reduction compare to 20% PEG treatment (Figure 3C).

### Physiological Responses

**FIGURE 4:**
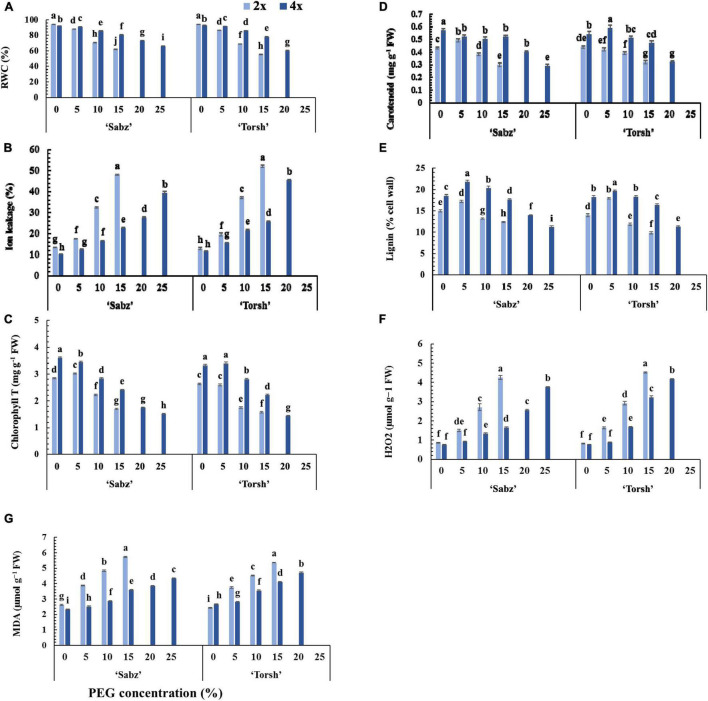
Physiological responses of diploid and tetraploid explants of ‘Sabz’ and ‘Torsh’ fig cultivars, 14 days after subjecting to different water stress treatments. **(A)** Relative water content, **(B)** ion leakage, **(C)** total chlorophyll content, **(D)** total carotenoid content, **(E)** lignin, **(F)** hydrogen peroxide, and **(G)** malondialdehyde. Means represent the ploidy level and PEG treatment effects tested by two-way ANOVA. Each value represents the means ± SE. Different letters indicate significant differences at *P* < 0.05 using the LSD test.

The results demonstrated that the RWC and ion leakage of tetraploid and diploid explants of both cultivars were differently affected by water stress treatments. Indeed, in tetraploid explants, decrement in RWC and increment in ion leakage occurred gradually following an increase in water stress intensity from the control level to 20 and 25% PEG treatments. In contrast, their diploid control explants displayed a drastic RWC decrement and ion leakage increment at 10% PEG treatment (Figures 4A,B). At 15% PEG treatment, the RWC was 1.3-fold and 1.4-fold higher in tetraploid explants of ‘Sabz’ and ‘Torsh’, respectively, compared to their diploid counterparts (Figure 4A). Additionally, at 20% PEG treatment, the RWC in ‘Sabz’ tetraploids was 20% higher than ‘Torsh’ tetraploids, and at 25% PEG treatment, the RWC in ‘Sabz’ tetraploids was placed on the critical range of 66.25%, while ‘Torsh’ tetraploid explants did not survive at this level of water stress (Figure 4A).

The ion leakage in ‘Sabz’ and ‘Torsh’ tetraploid genotypes was 2.1- and 2-fold, respectively, less than their diploid control plants at 15% PEG treatment. Also, the ion leakage of ‘Sabz’ tetraploids (27.52%) was significantly less than ‘Torsh’ tetraploids (45.22%) at 20% PEG treatment. Furthermore, ion leakage of ‘Sabz’ tetraploids reached its highest percent (39.25%) at 25% PEG treatment, which was a 29.8% increase compared with 20% PEG treatment (Figure 4B).

According to the results, increasing water stress intensity significantly decreased the content of the photosynthetic pigments (total chlorophyll and carotenoids) in all genotypes, however, the carotenoid content was less affected by the detrimental effects of water stress treatments than chlorophyll (Figures 4C,D). With increasing water stress intensity, both tetraploid genotypes showed a higher ability for the biosynthesis of photosynthetic pigments. Therefore, at 15% PEG treatment, the chlorophyll content was found significantly 1.4-fold higher in both tetraploid explants than their diploid control plants. At 20% PEG treatment, tetraploid explants of ‘Sabz’ had significantly higher chlorophyll content (1.75 mg g^–1^ FW) than tetraploid explants of ‘Torsh’ (1.44 mg g^–1^ FW). The chlorophyll content of ‘Sabz’ tetraploids was 1.51 mg g^–1^ FW, at 25% PEG treatment, displaying a 13.7% defacement than 20% PEG treatment (Figure 4C).

The carotenoid content was also significantly higher and up to 42 and 31% in tetraploid explants of ‘Sabz’ and ‘Torsh’, respectively, than their diploid controls at 15% PEG treatment. Besides, compared to tetraploid explants of ‘Torsh’ (0.32 mg g^–1^ FW), the carotenoid content was significantly higher in tetraploid explants of ‘Sabz’ (0.4 mg g^–1^ FW) at 20% PEG treatment. The carotenoid content of ‘Sabz’ tetraploids showed a decrease of 27.5% at 25% PEG treatment compared to 20% PEG treatment (Figure 4D).

The lignin biosynthesis in all genotypes was enhanced at 5% PEG treatment, and then it decreased by increasing PEG concentrations. The lignin content was significantly higher up to 1.4-fold and 1.6-fold in tetraploid explants of ‘Sabz’ and ‘Torsh’ cultivars, respectively, than their diploid control plant. At 20% PEG treatment, ‘Sabz’ tetraploids synthesized significantly more lignin (14% cell wall) than tetraploid explants of ‘Torsh’ (11.3% cell wall). Under 25% PEG treatment, tetraploids explants of the ‘Sabz’ cultivar showed 11.3% lignin cell wall (Figure 4E). The results showed that H_2_O_2_ and MDA contents were significantly amassed in all genotypes by increasing water stress intensity. The H_2_O_2_ and MDA contents were notably lower in both tetraploid genotypes than their diploid control plants at 5, 10, and 15% PEG treatments (Figures 4F,G). At 15% PEG treatment, the H_2_O_2_ content was 2.5-fold and 1.4-fold lower in tetraploid explants of ‘Sabz’ and ‘Torsh’ cultivars, respectively, compared to their diploid control plants. Furthermore, the H_2_O_2_ content of tetraploid explants of ‘Sabz’ was significantly lower (1.6-fold) than tetraploid explants of ‘Torsh’ at 20% PEG treatment. The H_2_O_2_ content in tetraploid explants of ‘Sabz’ showed a 47.2% increase and reached 3.77 μmol g^–1^ FW at 25% PEG treatment (Figure 4F).

Polyethylene glycol treatments increased the MDA content of all genotypes, similar to the trend of H_2_O_2_ content in a concentration-dependent manner. At 15% PEG treatment, the MDA content of tetraploid explants of ‘Sabz’ and ‘Torsh’ cultivars was significantly lower (1.6-fold and 1.3-fold, respectively) than their diploid control plants. The MDA content in tetraploid explants of the ‘Sabz’ cultivar (3.84 μmol g^–1^ FW) was significantly lower than tetraploid explants of the ‘Torsh’ cultivar (4.71 μmol g^–1^ FW) at 20% PEG treatment. Also, the MDA content in tetraploid explants of ‘Sabz’ cultivar with a gradual increase reached 4.34 μmol g^–1^ FW at 25% PEG treatment (Figure 4G).

### Biochemical Responses

**FIGURE 5:**
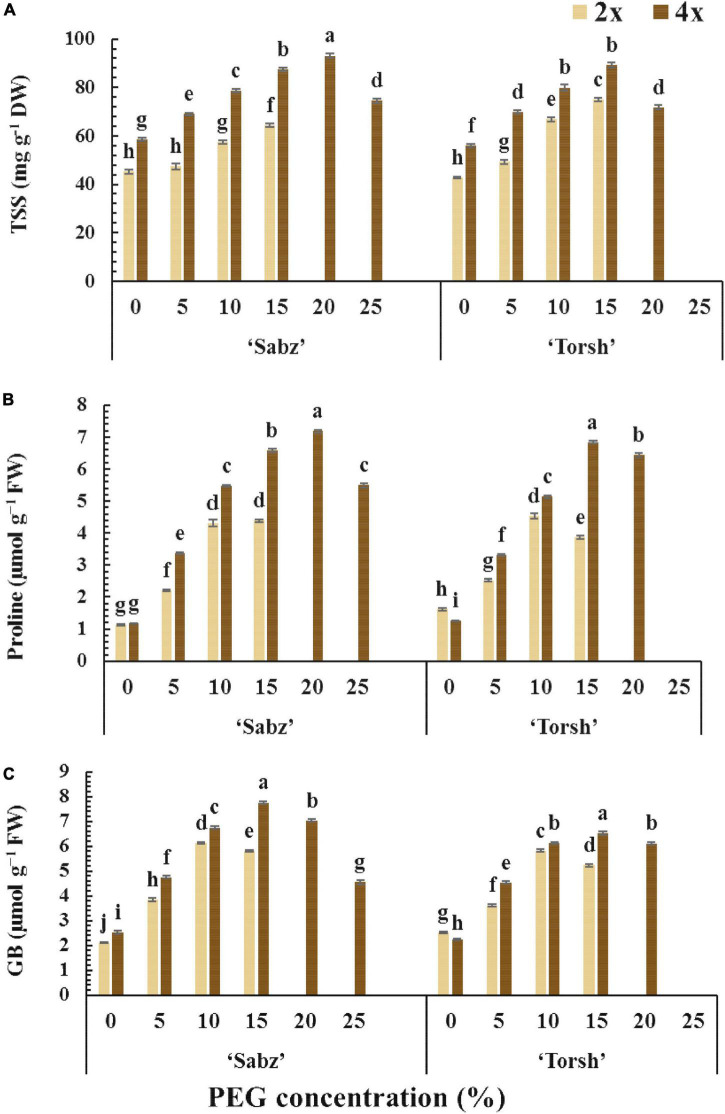
The comparison of non-enzymatic defense system responses of tetraploid and their diploid control explants of ‘Sabz’ and ‘Torsh’ fig cultivars, 14 days after subjecting to different water stress treatments. **(A)** Total soluble sugars, **(B)** proline, and **(C)** glycine betaine. Means represent the ploidy level and PEG treatment effects tested by two-way ANOVA. Each value represents the means ± SE. Different letters indicate significant differences at *P* < 0.05 using the LSD test.

The TSS accumulation was greatly increased by increasing water stress treatments in all genotypes compared to their control treatments. Tetraploid genotypes of both cultivars (‘Sabz’ and ‘Torsh’) exhibited significantly higher TSS accumulated by 1.35-fold and 1.2-fold, respectively, than their diploid control genotypes, under 15% PEG treatment. At 20% PEG treatment, the TSS content of ‘Sabz’ tetraploids was significantly higher (up to 1.3-fold) than ‘Torsh’ tetraploids, and at 25% PEG treatment, ‘Sabz’ tetraploids had 74.55 mg g^–1^ DW of TSS that decreased by 19% than its content under 20% PEG treatment (Figure 5A).

The accumulation of proline and glycine betaine (GB) displayed a similar rising trend in all genotypes with increasing PEG concentrations. However, their accumulations were significantly higher in tetraploid genotypes than their diploid control plants at 5, 10, and 15% PEG treatments (Figures 5B,C). At 15% PEG treatment, the proline content of ‘Sabz’ and ‘Torsh’ tetraploids was 1.5-fold and 1.7-fold higher than their diploid control plants, respectively. In addition, at 20% PEG treatment, the proline content of ‘Sabz’ tetraploids was significantly greater (11% higher) than ‘Torsh’ tetraploids. The proline content of ‘Sabz’ tetraploids was 5.49 μmol g^–1^ FW at 25% PEG treatment, showing a 23% decrease than 20% PEG treatment (Figure 5B).

While the GB content of both tetraploid genotypes showed an increasing trend from the control to 15% PEG treatment, this trend was observed only in 5 and 10% PEG treatments in their diploid control genotypes. The GB content of ‘Sabz’ and ‘Torsh’ tetraploids was significantly higher (1.33-fold and 1.25-fold, respectively) than their diploid controls at 15% PEG treatment. At 20% PEG treatment, the GB content of ‘Sabz’ tetraploids was significantly higher (13% higher) than ‘Torsh’ tetraploids. Also, the GB content of ‘Sabz’ tetraploids was 4.55 μmol g^–1^ FW at 25% PEG treatment, which showed a 35% decrease than 20% PEG treatment (Figure 5E).

### Enzymatic Activities

**FIGURE 6:**
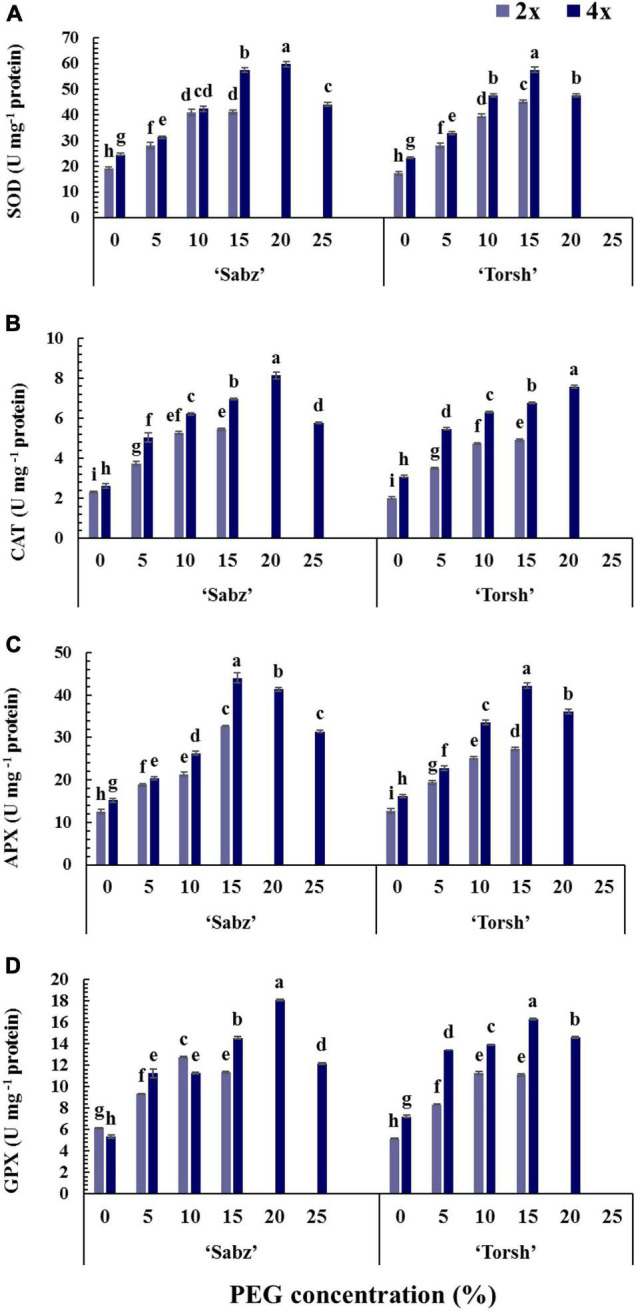
Enzymatic activity responses of diploid and tetraploid explants of ‘Sabz’ and ‘Torsh’ fig cultivars 14 days after subjecting to different water stress treatments. **(A)** Superoxide dismutase, **(B)** catalase, **(C)** ascorbate peroxidase, and **(D)** glutathione peroxidase. Means represent the ploidy level and PEG treatment effects tested by two-way ANOVA. Each value represents the means ± SE. Different letters indicate significant differences at *P* < 0.05 using the LSD test.

The enzymatic defense system, including the activity of SOD, CAT, APX, and GPX enzymes, was significantly enhanced with increasing water stress intensity in all genotypes (Figure 6). The results demonstrated that at 5, 10, and 15% PEG treatments, antioxidant enzymes activities were significantly higher in both tetraploid genotypes compared to their diploid control plants. Accordingly, at 15% PEG treatment, the SOD activity in ‘Sabz’ and ‘Torsh’ tetraploids was 1.4-fold and 1.27-fold higher than their diploid control explants, respectively. SOD activity was significantly higher (1.25-fold) in ‘Sabz’ tetraploids compared to ‘Torsh’ tetraploids at 20% PEG treatment. At 25% PEG treatment, SOD activity was 44.18 U mg^–1^ protein in ‘Sabz’ tetraploids, showing a 26% decrease than its activity at 20% PEG treatment (Figure 6A).

Catalase activity showed a 1.27-fold and 1.37-fold increase in ‘Sabz’ and ‘Torsh’ tetraploid genotypes compared to their diploid control genotypes under 15% PEG treatment. In addition, CAT activity in ‘Sabz’ tetraploids was significantly higher (6% higher) than ‘Torsh’ tetraploids at 20% PEG treatment. At 25% PEG treatment, CAT activity in the ‘Sabz’ tetraploids was 5.78 U mg^–1^ protein, which displayed a 28% decrease compared to the 20% PEG treatment (Figure 6B).

Ascorbate peroxidase activity was also significantly higher in ‘Sabz’ and ‘Torsh’ tetraploids by 1.34-fold and 1.54-fold, respectively, than their diploid control explants at 15% PEG treatment. At 20% PEG treatment, the APX activity of ‘Sabz’ tetraploids was significantly higher (up to 12.5%) than ‘Torsh’ tetraploids. Also, the APX activity in ‘Sabz’ tetraploids was 31.45 U mg^–1^ protein at 25% PEG treatment, significantly lower than its activity at 20% PEG treatment (Figure 6C).

Both ‘Sabz’ and ‘Torsh’ tetraploid genotypes showed a significantly greater GPX activity (1.28-fold and 1.46-fold, respectively) at 15% PEG treatment than their diploid control genotypes. Also, the GPX activity was significantly higher (19% higher) in ‘Sabz’ tetraploids than ‘Torsh’ tetraploids at 20% PEG treatment. GPX activity in ‘Sabz’ tetraploids was 12.19 U mg^–1^ protein at 25% PEG treatment and was significantly lower than its activity under 20% PEG treatment (Figure 6D).

## Discussion

Under natural conditions, the plant roots have a determinant role in plant water stress responses. Nevertheless, the shoot tip explants (without roots) have proved to be a good representative for an entire plant under *in vitro* conditions for abiotic stress studies, including drought and salinity (Peìrez-Clemente and Gomez-Cadenas, 2012). In this regard, shoot tip explants have recently been widely used to study various aspects of plant strategies encountering water stress conditions in many plants’ species *in vitro*. As reported in *Eucalyptus tereticornis* (Singh et al., 2020), *Actinidia* spp. (Zhong et al., 2018), *Lippia alba* (De Castro et al., 2020), *Prunus* spp. (Sorkheh et al., 2011), etc. A shoot tip explant behaves like an entire plant under water stress conditions and may indicate the water stress coping mechanisms in the roots and shoots, including rapid hormonal responses, osmoprotectants accumulation, enzymatic and non-enzymatic defensive strategies (Montoliu et al., 2009; Peìrez-Clemente and Gomez-Cadenas, 2012).

Water stress significantly limits plant growth and development by affecting different morphological, hormonal, physiological, and biochemical aspects (Khalid et al., 2020). *In vitro* water stress is usually accompanied by a typical range of symptoms. In the current study, depending on genotype, various levels of leaf wilting and rolling, yellowing and necrosis of the leaf margins, leaf shedding, shoot tip dieback, and stem browning were observed by the increased water stress intensity and duration. The observations pointed that both cultivars’ tetraploid explants were more resilient to the high level of water stress treatments (15, 20, and 25% PEG treatments) than their diploid control explants (Figure 2). For example, at 15% PEG treatment, both diploid genotypes showed severely damaged symptoms, including drastic leaf shedding, decreasing leaf area, leaf necrosis, stem browning, shoot tip dieback, as previously reported (Karimi et al., 2012; Shekafandeh and Hojati, 2013). In both tetraploid genotypes, the symptoms were less severe and limited to reduced growth, leaf numbers and area, and some degree of yellowish.

The results highlighted the superiority of both tetraploid genotypes in all tested morphological parameters at different water stress treatments than their diploid control plants (Figure 2). This indicates that tetraploid genotypes benefited from the adaptive strategies that alleviate the detrimental effects of water stress, especially at high levels of PEG treatments (15, 20, and 25% PEG concentrations).

In plants, water stress tolerance is highly related to the modulating and coordinating gene expression networks, especially regarding turgor maintenance, preventing cell dehydration, and cell detoxification. Various physiological and biochemical strategies are involved (Pandey and Chakraborty, 2015; Hasanuzzaman et al., 2020). As Zhang et al. (2015) reported, these strategies resulted in superior drought tolerance of two tetraploid apple cultivars (‘Hanfu’ and ‘Gala’) compared to their diploid seedlings under short term extreme drought conditions (30% PEG).

Rapid phytohormonal changes follow the formation of the first hyperosmotic signals in plant cells under water stress conditions. Drought adaptive strategies, including morphological, physiological, and biochemical modifications, are then regulated by the phytohormonal responses (Zhu, 2016). In this regard, ABA, SA, and JA are considered major stress response phytohormones (SRHs) (Yu et al., 2020). The biosynthesis, and accumulation of key SRH, ABA, increase rapidly by receiving the first hyperosmotic signals and regulating a series of downstream genes, especially overexpression of genes involving the compatible solutes, osmoprotectants, and enzymatic and non-enzymatic defensive system. ABA also regulates the closure of stomata to reduce transpiration (Pandey and Chakraborty, 2015).

The hormonal behavior analysis in this study revealed that ABA biosynthesis in tetraploid explants of both cultivars was around twofold higher than their diploid control plants under moderate and high water-stress levels. The SA and JA levels and their biosynthesis were significantly higher in tetraploid explants at the high water-stress levels than in their diploid control plants. They synergistically work with ABA to alleviate detrimental effects of water stress by triggering the downstream mechanisms, including rapid stomatal closure, over-accumulation of compatible solutes and osmoprotectants, over-activating the enzymatic antioxidant system, and increasing hydraulic conductivity of water to leaves at stressful times (Wager and Browse, 2012; Sánchez-Romera et al., 2014). Herein, 20% (severe) and 25% (extreme) PEG treatments negatively affected ABA, SA, and JA biosynthesis of tetraploid genotypes, which indicated that very high water-stress intensities could seriously disrupt their biosynthesis pathways.

Enhanced efficiency of adaptive strategies under water stress, including robust osmotic adjustment and enzymatic defense system, all influenced by augmented ABA signaling pathways as a result of chromosome doubling, have been well investigated (Allario et al., 2013; Rao et al., 2020). However, there is not enough information about the molecular mechanisms underlying this phenomenon. Rao et al. (2020), by comparing transcriptional analysis of autotetraploid and diploid plants of *L. ruthenicum*, discovered that the substantial superiority of autotetraploids over diploid plants is mainly related to their intrinsic capability to rapidly increase ABA biosynthesis and accumulation *via* overexpression of *9-cis-epoxycarotenoid dioxygenase 1 (NCED1)* and *NCED2*, which encode pivotal biosynthetic enzymes in ABA signaling transduction pathways. Additionally, Allario et al. (2013) reported that tolerance to water stress of 4× Rangpur lime rootstocks than their 2× counterparts is because of their boosted ability for ABA biosynthesis and ABA regulatory system, which is regulated by overexpression of drought-responsive genes, including *CsNCED1*, a critical regulatory gene of ABA biosynthesis.

Increasing ion leakage is an essential index for cell membrane stability under stressful conditions, reported in all types of abiotic stresses (Santos et al., 2019). The significantly low percentage of ion leakage in tetraploid explants than their diploid control plants in the present study might be related to their superior adaptive strategies such as robust osmotic adjustment and cell detoxification systems, which lead to maintaining the cell membrane integrity and preventing its destruction (Wei et al., 2019). These augmented critical strategies resulting from chromosome doubling could also play a key role in preventing the degradation of photosynthetic pigments and plant cytokinins, which are essential for photosynthetic pigments biosynthesis (Du et al., 2020). Due to that, tetraploid explants had significantly greater chlorophyll and carotenoid contents under all water stress treatments. However, the tetraploid figs essentially produced much more photosynthetic pigments at the non-stress condition (Figures 4C,D).

The tetraploid explants of both cultivars contained significantly more lignin in their cell walls than their diploid control plants. Lignin content increased only at 5% PEG treatment in diploids genotypes, while it increased even at 10% PEG treatment in tetraploid genotypes compared to their control treatment. Although the lignin content declined for both tetraploid and diploid explants at higher water stress levels, the decline rate was quicker for diploids (Figure 4E). The increase in lignin biosynthesis is due to overexpression of *MdMYB4* and *MdVND6* genes, which are two essential genes in lignin biosynthesis pathways, resulting in lignin deposition, xylem vessel synthesis, better hydraulic conductivity, more cell wall, and membrane stability, that all lead to increasing drought adaptation (Geng et al., 2018; Xu et al., 2020). Our data support previous work across plant species that showed that severe abiotic stress, including water stress, can limit lignin biosynthesis (Moura-Sobczak et al., 2011). A decrement in the biosynthesis of lignin precursors such as caffeoyl-CoA and para-coumaryl alcohol and declined anionic peroxidase activity, have been reported as the possible reasons for lignin biosynthesis suppression (Vincent et al., 2005; Moura et al., 2010).

H_2_O_2_ and MDA are two well-known stress biomarkers in plant systems. Excessive water loss in plants under drought conditions gives rise to over-producing ROSs, which damage cell membranes and increase ion leakage. Increasing MDA content indicates enhanced lipid peroxidation of the cell membrane. The greater ability of tetraploid genotypes for osmotic adjustment and their robust ROS scavenging systems significantly reduces their H_2_O_2_ and MDA contents under high-level water stress treatments (Jaafar et al., 2012).

Osmotic adjustment through synthesis and accumulation of compatible solutes is one of the most critical plant strategies confronting water stress conditions. Compatible solutes mostly include soluble sugars (TSSs), amino acids (especially proline), and quaternary ammonium compounds (notably GB), which are rich in hydroxyls (−OH) groups that allow them to bind to H_2_O molecules and protect cellular ingredients from dehydration, keeping vital functions of proteins and enzymes and preserving cell structures and membranes (Laxa et al., 2019; Hasanuzzaman et al., 2020).

As the results revealed, tetraploid genotypes in this study displayed a greater intrinsic capability for cell osmolytes accumulation than their diploid controls. This essential superiority was clearly observed (for only ‘Sabz’ tetraploids) at severe and extreme water stress conditions (20 and 25% PEG concentrations), where the tetraploid genotypes were capable of biosynthesis and accumulating a remarkable amount of cell osmolytes whilst their diploid controls did not tolerate such a level of water stress and died (Figure 5). Cell osmolytes accumulation, including TSS, proline, and GB, is well known as a central plant’s strategy for confronting water stress, which functionally protects cell ingredients from excessive dehydration, as well as less generation of ROSs, ROS scavenging, and preventing cell membrane disruption (Gurrieri et al., 2020). It could be considered as a significant strategy in which tetraploid genotypes cope with water stress and display extra tolerance to severe water stress conditions compared with their diploid genotypes. Our findings support previous reports that enhanced osmolyte accumulation confers superiority to tetraploid genotypes to better tolerate water stress than diploids (Liu et al., 2011; Zhang et al., 2015; Wei et al., 2019; Oustric et al., 2020).

Stomatal closure, as one of the earliest plant responses to water stress conditions, leads to the disrupting of the thylakoid electron transport chain, which results in the overaccumulation of various forms of ROSs in cell organelles such as chloroplasts, mitochondria, peroxisomes, as well as apoplast, and plasma membranes (Laxa et al., 2019). Due to their strong tendency to react with cellular ingredients such as carbohydrates, proteins, lipids, nucleic acids, cell membranes, etc., the ROS molecules are highly detrimental for plant systems and need to be rapidly removed. Plants counteract ROSs by boosting enzymatic (SOD, CAT, APX, GPX) and non-enzymatic (phenolic compounds, non-protein amino acids) defensive systems to maintain the steady-state balance in cells (Hasanuzzaman et al., 2020). As displayed in Figure 6, tetraploid explants benefited from an augmented antioxidant defense system under water stress treatments compared to their diploid control plants. Obviously, under the high levels of water-stress condition, the significant superiority of SOD, CAT, APX, and GPX activity in tetraploid explants resulted in robust ROS scavenging and cell detoxification than their diploid control plants. The antioxidant enzymes act together synergistically, in a so-called chain reaction for cell protection against ROSs toxicity (Su et al., 2017). The enhanced enzymatic antioxidant system resulting from the chromosome doubling in the current research were also reported in autotetraploid plants of *P. trifoliata* (Wei et al., 2019), ‘Carrizo’ citrange (Oustric et al., 2017), *Dioscorea zingiberensis* (Zhang et al., 2010), and *Robinia pseudoacacia* (Wang et al., 2013).

The vital function of the enzymatic defense system briefly includes catalyzing the conversion of two molecules of O_2_^•–^ into H_2_O_2_ and O_2_ by SOD enzyme and its metal isoforms (Mn-SOD, Fe-SOD, Cu, Zn-SOD, and Ni-SOD). This is followed by decomposing H_2_O_2_ into H_2_O and O_2_ by CAT, APX, GPX enzyme activities *via* mediating different substrates to protect cellular ingredients from the ROSs injuries (Mittler, 2002; Rai et al., 2011; Santos et al., 2019; Zhang et al., 2019).

## Conclusion

In the present study, tetraploid-induced explants of two fig cultivars (‘Sabz’ and ‘Torsh’) and their diploid control plants were subjected to different *in vitro* PEG-induced water stress treatments (0, 5, 10, 15, 20, and 25%) to evaluate their water stress tolerance. The morphological, hormonal, physiological, and biochemical analysis revealed that both tetraploid genotypes exhibited superior water stress tolerance than their diploid control plants. Among the four tested genotypes, the tetraploid explants of the ‘Sabz’ cultivar were the only genotype that could withstand the extreme (25% PEG) water stress conditions. Tetraploid plantlets of both cultivars displayed a superior ability for maintaining their RWC and ion leakage percentages in a low-risk range compared with their diploid genotypes. They also benefit from augmented adaptive strategy systems, including remarkably higher biosynthesis of ABA, SA, and JA stress response hormones, robust osmotic adjustment (higher accumulated TSS, proline, and glycine-betaine), and a strong enzymatic defense system (notably higher SOD, CAT, APX, and GPX activities). Our results paved the way for using the ploidy manipulation method in fig tree breeding programs as an efficient breeding system to achieve new desirable characteristics with significant tolerance to abiotic stresses and to overcome the conventional breeding system’s barriers of the fig tree.

## Data Availability Statement

The original contributions presented in the study are included in the article/Supplementary Material, further inquiries can be directed to the corresponding author.

## Author Contributions

RA conducted the experiments, data analyzing, and interpretation, and wrote the manuscript. AS contributed to the supervisor, research design, assistance with data interpretation, correction, and approval of the manuscript. Both authors contributed to the article and approved the submitted version.

## Conflict of Interest

The authors declare that the research was conducted in the absence of any commercial or financial relationships that could be construed as a potential conflict of interest.

## Publisher’s Note

All claims expressed in this article are solely those of the authors and do not necessarily represent those of their affiliated organizations, or those of the publisher, the editors and the reviewers. Any product that may be evaluated in this article, or claim that may be made by its manufacturer, is not guaranteed or endorsed by the publisher.

## Abbreviations

PEG: polyethylene glycol
ABA: abscisic acid
SA: salicylic acid
JA: jasmonic acid
UPLC-MS: ultra performance liquid chromatography–mass spectrometer.
