# Supplementary material for: Tetraploidy Confers Superior in vitro Water-Stress Tolerance to the Fig Tree (Ficus carica) by Reinforcing Hormonal, Physiological, and Biochemical Defensive Systems
Source: Front Plant Sci. 2022 Jan 28;12:796215. doi: 10.3389/fpls.2021.796215 (PMC8834540; doi:10.3389/fpls.2021.796215)
Supplement: Supplementary file 1 [file Table_1.docx]

**Supplementary data: ANOVA analysis**

| **Table S1** Analysis of variance (ANOVA) for the effect of 3 independent variables (PEG treatment, ploidy, and cultivar) on morphological, hormonal, physiological, and biochemical characteristics. | | | | | | | | |
| --- | --- | --- | --- | --- | --- | --- | --- | --- |
| **Source of variance** | **DF** | **Survival rate** | **Proliferation rate** | **Shoot length** | **Growth rate** | **Stem diameter** | **Leaf number** | **Total leaf area** |
|  |  | **Mean Square** | **Mean Square** | **Mean Square** | **Mean Square** | **Mean Square** | **Mean Square** | **Mean Square** |
| **Cultivar** | 1 | 3037.5000** | 2.1600000** | 6.5104167** | 0.00881667** | 0.2320667** | 77.041667** | 2247.69615** |
| **Ploidy** | 1 | 14504.1667** | 4.1666667** | 78.8437500** | 0.12906667** | 9.4000167** | 315.375000** | 3460.80167** |
| **WS** | 5 | 20254.1667** | 38.7449167** | 93.9506667** | 0.21032667** | 33.7388717** | 205.441667** | 9315.89005** |
| **Cultivar × Ploidy** | 1 | 1204.1667** | 0.6666667** | 3.3750000** | 0.00201667** | 0.7848167** | 45.375000** | 1784.68507** |
| **Cultivar × WS** | 5 | 727.5000** | 0.4962500** | 0.9859167** | 0.00040167 **ns** | 0.7819067** | 3.991667** | 139.57727** |
| **Ploidy × WS** | 5 | 3594.1667** | 4.5569167** | 4.1017500** | 0.00994167** | 2.4527867** | 26.425000** | 237.81745** |
| **Cultivar × Ploidy × WS** | 5 | 1094.1667** | 0.3229167** | 1.3925000** | 0.00078667 **ns** | 0.6102617** | 2.975000** | 194.62471** |
| **Error** | 72 | 51.3889 | 0.010208 | 0.062361 | 0.0004388 | 0.011766 | 0.7777 | 23.7394 |
| **CV** |  | 9.74 | 10.18 | 6.26 | 18.69 | 4.51 | 21.27 | 21.12 |
| **Source of variance** | **DF** | **Fresh weight** | **Dry weight** | **ABA** | **SA** | **JA** | **Water content** | **Ion leakage** |
|  |  | **Mean Square** | **Mean Square** | **Mean Square** | **Mean Square** | **Mean Square** | **Mean Square** | **Mean Square** |
| **Cultivar** | 1 | 11.3712667** | 1.5990844** | 64.016867** | 11517.6491** | 1661.08801** | 1329.82594** | 0.33844 **ns** |
| **Ploidy** | 1 | 50.1126000** | 5.1198844** | 939.763865** | 59449.2696** | 20730.2365** | 12841.3134** | 42.00260** |
| **WS** | 5 | 195.779652** | 20.1770494** | 494.451285** | 34848.1956** | 6047.55931** | 15674.8071** | 1676.99635** |
| **Cultivar × Ploidy** | 1 | 7.3593375** | 0.7297594** | 12.402750** | 6205.4328** | 1395.75628** | 807.94010** | 63.53760** |
| **Cultivar × WS** | 5 | 2.0231917** | 0.1835044** | 8.976043** | 986.1022** | 630.48856** | 660.21119** | 405.84069** |
| **Ploidy × WS** | 5 | 4.0238750** | 0.5072644** | 113.305350** | 5456.8887** | 2157.52624** | 2435.46019** | 2103.87835** |
| **Cultivar × Ploidy × WS** | 5 | 1.5855125** | 0.1989794** | 17.092658** | 1459.4219** | 673.28480** | 752.70585** | 359.15235** |
| **Error** | 72 | 0.08949 | 0.017955 | 0.009891 | 0.0652 | 0.06841 | 0.4816 | 0.71094 |
| **CV** |  | 5.96 | 8.2 | 1.1 | 0.3 | 0.56 | 10.9 | 4.21 |
| **Source of variance** | **DF** | **Total chlorophyll** | **Carotenoid** | **Lignin** | **H_2_O_2_** | **MDA** | **TSS** | **Proline** |
|  |  | **Mean Square** | **Mean Square** | **Mean Square** | **Mean Square** | **Mean Square** | **Mean Square** | **Mean Square** |
| **Cultivar** | 1 | 2.1182042** | 0.0228166** | 95.20166** | 0.0155041 **ns** | 1.1682094** | 954.07260** | 5.645400** |
| **Ploidy** | 1 | 17.992016** | 0.7072666** | 966.4704** | 1.0626041** | 2.943501** | 24008.435** | 127.99249** |
| **WS** | 5 | 21.573706** | 0.5266675** | 663.3794** | 15.220276** | 27.014841** | 7510.8612** | 47.325680** |
| **Cultivar × Ploidy** | 1 | 0.2185042** | 0.0204166** | 40.04166** | 0.112066** | 0.066676** | 2164.4802** | 7.7668504** |
| **Cultivar × WS** | 5 | 0.2084742** | 0.0141641** | 13.46716** | 4.109919** | 3.7344194** | 1070.4152** | 5.1241065** |
| **Ploidy × WS** | 5 | 0.535456** | 0.0388841** | 45.28041** | 16.51181** | 22.950221** | 2749.6922** | 24.298615** |
| **Cultivar × Ploidy × WS** | 5 | 0.501264** | 0.0201641** | 20.66016** | 3.709056** | 4.355616** | 798.66047** | 4.8425634** |
| **Error** | 72 | 0.004279 | 0.000704 | 0.23722 | 0.01189 | 0.00457 | 2.30052 | 0.008139 |
| **CV** |  | 3.32 | 7.52 | 3.9 | 6.37 | 2.3 | 2.85 | 2.81 |
| **Source of variance** | **DF** | **Glycine-betaine** | **SOD** | **CAT** | **APX** | **GPX** |  |  |
|  |  | **Mean Square** | **Mean Square** | **Mean Square** | **Mean Square** | **Mean Square** |  |  |
| **Cultivar** | 1 | 12.098400** | 424.11634** | 8.5085042** | 138.72041** | 20.01113** |  |  |
| **Ploidy** | 1 | 94.923037** | 7296.8962** | 170.77335** | 4242.7004** | 656.312** |  |  |
| **WS** | 5 | 69.519405** | 3270.2846** | 50.340089** | 1588.730** | 247.7996** |  |  |
| **Cultivar × Ploidy** | 1 | 8.4135042** | 444.7926** | 2.6070042** | 123.3066** | 2.05627** |  |  |
| **Cultivar × WS** | 5 | 2.777230** | 343.8468** | 5.2131417** | 212.4596** | 29.28002** |  |  |
| **Ploidy × WS** | 5 | 22.743342** | 1459.9308** | 28.060467** | 737.0821** | 136.9459** |  |  |
| **Cultivar × Ploidy × WS** | 5 | 2.835814** | 342.8412** | 6.528446** | 184.0769** | 39.5600** |  |  |
| **Error** | 72 | 0.009802 | 1.9513 | 0.025385 | 0.8334 | 0.0520 |  |  |
| **CV** |  | 2.52 | 4.59 | 3.97 | 4.38 | 2.56 |  |  |
| WS water stress. CV coefficient of variation. ** Significance and ^ns^ Non significance at the 5% level probability. | | | | | | | | |
